# Clinical characteristics of 31 hemodialysis patients with 2019 novel coronavirus: a retrospective study

**DOI:** 10.1080/0886022X.2020.1796705

**Published:** 2020-07-28

**Authors:** Jiong Zhang, Fang Cao, Shu-Kun Wu, Lu Xiang-Heng, Wei Li, Gui-sen Li, Jia Wang

**Affiliations:** a Department of Nephrology, University of Electronic Science and Technology, Sichuan Academy of Sciences & Sichuan Provincial People's Hospital, Sichuan Clinical Research Center for Kidney Disease, Chengdu, China; b Department of Nephrology, The First People's Hospital of Liangshan Yi Autonomous Prefecture, Xichang, China; c Queen Mary Colleges, Medical College of Nanchang University, Nanchang, China; d Department of Nephrology, Wuhan Fourth Hospital, Puai Hospital, Tongji Medical College, Huazhong University of Science and Technology, Wuhan, China; e Department of General Medicine Center, University of Electronic Science and Technology, Sichuan Academy of Sciences & Sichuan Provincial People's Hospital, Chengdu, China

**Keywords:** Hemodialysis, clinical characteristics, COVID-19, epidemiology, chronic inflammation

## Abstract

**Aim:**

Novel coronavirus pneumonia (COVID-19) has become pandemic. It brings serious threat to hemodialysis (HD) patients. Therefore, we carried out a study on the clinical characteristics of HD patients with COVID-19.

**Methods:**

We retrospectively analyzed the data of 31 HD patients with COVID-19. The clinical features of patients include epidemiology, clinical symptoms, laboratory and imaging test, treatment and prognosis.

**Results:**

61.3% were severe, and 38.7% were mild. 83.9% had a close contact history with COVID-19 patients. The average age was 62.3 years comprising of 58.1% men and 41.9% women. Ninety percent had chronic diseases except ESRD. Clinical symptoms include cough (85%), fever (43%), and shortness of breath (48.4%), etc. Complications included ARDS (25.8%), AHF (22.6%), and septic shock (16.1%), etc. 64.5% of patients had remission, and 35.5% of patients had no remission with 6.5% deaths. Compared with the baseline before infection, HD patients with COVID-19 had lower lymphocytes, albumin and glucose, and higher D-dimer, albumin, phosphorus, lactate dehydrogenase, and CRP. There was no significant correlation between the neutrophils/lymphocytes ratio and the severity of the disease.

**Conclusions:**

Compared with the reported general population, the HD patients are susceptible to COVID-19 infection, especially older men and those with other underlying diseases. Moreover, HD patients have more severe infection and inflammation with less symptoms and worse outcome. COVID-19 infection can cause dialysis patients lower immunity, stronger inflammation, malnutrition, and internal environment disorder. Neutrophils/lymphocytes ratio does not reflect the severity of the HD patients with COVID-19.

## Introduction

Novel coronavirus pneumonia (COVID-19) has become a catastrophic pandemic due to high pathogenicity and susceptibility. Therefore, it is a serious threat to human life, health, and property, especially for patients who have underlying diseases such as diabetes, renal failure, tumor, heart and brain diseases, etc. [1]. These patients are weaker and more susceptible to infection than normal people [2,3]. It is necessary to pay more attention on these patients. Unfortunately, there are few studies on the clinical characteristics of hemodialysis (HD) patients with COVID-19 to date. Given the huge population base of HD patients worldwide, the high infectivity of COVID-19 and the weak immunity and susceptibility of HD patients, we retrospectively studied the clinical characteristics of HD patients with COVID-19 in Wuhan.

## Methods

### Research target

We assessed a total of 31 HD patients with COVID-19 in Wuhan Fourth Hospital. It is where the first batch of designated hospitals for patients were infected with COVID-19. The patients were transferred from the different dialysis centers after being diagnosed with COVID-19 infection by COVID-19 expert group on the base of WHO interim guidance [4]. The study protocol was authorized and approved by the Ethics Committee of Sichuan Provincial People's Hospital. The approval number is 2020-113.

### Data collection

All medical records of patients in this study were carefully analyzed by the researchers. The collecting clinical information containing the medical history, epidemiology, sign, symptoms, chest CT scan, blood routine test, underlying diseases, treatment and prognosis was obtained through the hospital's electronic medical record system. These data were repeatedly reviewed by a team of professionally trained doctors.

### Statistical analysis

Continuous variables were illustrated as median (IQR) and compared with the *t*-test; enumeration data were represented as number (%). Correlation between neutrophils/lymphocytes ratio and disease severity is analyzed by using Spearman’s correlation. All data statistical analyses were carried out by using SPSS 12.0 (SPSS Inc., Chicago, IL), and the value (*p* < .05) is regarded as statistically significantly different.

## Results

### Basic information

In the study, the vascular access patterns of 31 patients were arteriovenous internal fistula (66.7%), semi-permanent catheters (29%), and temporary catheter (3.2%). The dialysis vintage of these patients was more than 5 years (41.9%), 1–5 years (51.6%), and less than 1 year (6.5%). The dialysis frequencies were two (74.2%) or three (25.8%) times a week at outpatient department and two (6.5%) or three (93.5%) times a week in hospital with 3 h for each dialysis (Table 1).

**Table 1.** Basic information of HD patients with COVID-19.

	Patients (*n* = 31)
Vascular access	
Semi-permanent catheters internal fistula	21 (67.7)
Semi-permanent catheters	9 (29)
Temporary catheter	1 (3.2)
Dialysis vintage (years)	
≥5	13 (41.9)
1–5	16 (51.6)
≤1	2 (6.5)
Dialysis frequency (outpatient)	
3 times a week	22 (71.0)
2 times a week	9 (29.0)
Dialysis frequency (hospital)	
3 times a week	29 (93.5)
2 times a week	3 (1.5–7)

### Clinical characteristics

Of the 31 patients, all of them are long-term maintenance HD patients in Wuhan with no history of seafood market exposure in South China in the last three months. But 83.9% of HD patients had a history of close contact with confirmed patients with COVID-19 infection. The average age of them was 62.3 years and 58.1% were males. Among them, 90% of these cases had other coexisting chronic diseases except ESRD, and the most common comorbidity was cardiovascular disease (including hypertension) (80.6%), followed by diabetes (19.4%) (Table 2). On admission, 61.3% of patients were severe, and 38.7% were mild according to clinical pulmonary infection score (CPIS). All patients received virus nucleic acid test with 38.7% positive results; 38.7% of patients had low fingertip oxygen saturation (Table 2).

**Table 2.** The clinical characteristics of patients infected with COVID-19.

Characteristics	Patients (*n* = 31)
Age, years	
Mean (SD)	62.3 (14.4)
Range	
≥70	9 (29)
60–69	10 (32.3)
50–59	6 (19.4)
40–49	4 (12.9)
≤39	2 (6.5)
Sex	
Women	13 (41.9)
Men	18 (58.1)
Contact history	26 (83.9)
Confirmed case	12 (38.7)
Chronic medical illness	
Hypertension	25 (80.6)
Diabetes	6 (19.4)
Cardiovascular disease	7 (22.6)
Malignancies	0 (0)
Pulmonary disease	1 (3.2)
Chronic liver disease	1 (3.2)
Grading of disease	
Mile	19 (61.3)
Severe	12 (38.7)
Critical	0 (0)
Confirmed case	12 (38.7)
Signs and symptoms at admission	
Fever	13 (41.9)
Highest temperature, °C	
<37·3	18 (58.1)
37·3–38·0	4 (12.9)
38·1–39·0	9 (29)
>39·0	0 (0)
Cough	26 (83.9)
Shortness of breath	15 (48.4)
Muscle ache	2 (6.5)
Headache	0 (0)
Sore throat	0 (0)
Rhinorrhea	0 (0)
Fatigue	11 (35.5)
Chest pain	2 (6.5)
Diarrhea	5 (16.1)
Nausea and vomiting	2 (6.5)
More than one sign or symptom	20 (64.5)
Fever, cough, and shortness of breath	7 (22.6)
Low oxygen saturation	12 (38.7)

### Laboratory results

The blood test results from three months ago were set as baseline and the onset of the illness is shown in Table 3. In total, 16 (51.6%) patients had lower baseline lymphocytes than the normal range. After infected with COVID-19, lymphocytes were significantly decreased. There was no significant change in neutrophils/lymphocytes ratios. Almost all patients (96.8%) had increased D-dimer. At the baseline, 24 (77.4%) patients had lower normal albumin level whereas 18 (58.1%) patients had higher blood phosphorus level, and 22 (71%) patients had higher glucose level than the normal range. After infection, albumin and glucose were significantly decreased, while blood phosphorus, lactate dehydrogenase, and CRP were significantly increased (Table 3).

**Table 3.** Laboratory findings of HD patients infected with COVID-19 on admission to hospital.

	Patients (*n* = 31)			
Blood routine	Baseline	Infection	统计量	*p* value
Leukocyte count (×10^9^ per L; normal range 3.5–9.5)	6.3 ± 2.4	5.4 ± 2.2	1.424	.165
Increased	1 (3.2)	1 (3.2)		
Decreased	0	5 (16.1)		
Neutrophils count (×10^9^ per L; normal range 1.8–6.3)	4.6 ± 1.9	4 ± 1.9	1.207	.237
Increased	2 (6.5)	4 (12.9)		
Decreased	0	3 (9.7)		
Lymphocytes count (×10^9^ per L; normal range 1.1–3.2)	1.1 ± 0.4	0.8 ± 0.3	2.407	.022*
Increased	0	0		
Decreased	16 (51.6)	25 (80.6)		
Neutrophils/lymphocytes ratios (n/L ratio)	4.3 (3.7–5.1)	4.9 (3.2–6.6)	–1.117	.264
Platelets (×10^9^ per L; normal range 125.0–350.0)	170.4 ± 55.5	198.6 ± 92.3	–1.515	.14
Increased	0	3 (9.7)		
Decreased	7 (22.6)	8 (25.8)		
Hemoglobin (g/L; normal range 115.0–150.0)	97.6 ± 15.7	92.4 ± 18.9	1.074	.291
Decreased	28 (90.3)	27 (87.1)		
Coagulation function				
D-dimer (µg/L; normal range 0.0–0.55)	0.3 (0.27–0.49)	1.8 (1.1–3.4)	–4.464	<.001**
Increased	9 (29)	30 (96.8)		
Blood biochemistry				
Albumin (g/L; normal range 40.0–55.0)	38.3 ± 2.1	33.4 ± 4.1	5.965	<.001**
Decreased	24 (77.4)	29 (93.5)		
Alanine aminotransferase (U/L; normal range 9.0–50.0)	10 (8–15.5)	10 (7–15.5)	–0.228	.82
Increased	2 (6.5)	2 (6.5)		
Aspartate aminotransferase (U/L; normal range15.0–40.0)	13 (9–17)	19 (14–23)	–2.224	.026
Increased	1 (3.2)	1 (3.2)		
Total bilirubin (μmol/L; normal range 0.0–21.0)	6.3 (4.7–7.4)	5.9 (4.5–7.2)	–1.261	.217
Increased	0	2 (6.5)		
Blood calcium (mmol/L; normal range 2.11–2.52)	2.1 ± 0.3	2.2 ± 0.2	–1.179	.247
Increased	1 (3.2)	1 (3.2)		
Decreased	16 (51.6)	13 (41.9)		
Blood phosphorus (mmol/L; normal range 0.97–1.6)	1.7 ± 0.5	2.3 ± 1	–2.473	.019*
Increased	18 (58.1)	27 (87.1)		
Decreased	0	1 (3.2)		
Blood urea nitrogen (mmol/L; normal range 3.6–9.5)	24.5 ± 5.7	28.4 ± 17	–1.304	.202
Increased	31 (100)	30 (96.8)		
Decreased	0	0 (0)		
Serum creatinine (μmol/L; normal range 57.0–111.0)	968.2 (774.5–1058.4)	903.5 (807.9–1282.2)	–0.451	.652
Increased	31 (100)	31 (100)		
Decreased	0	0		
Creatine kinase (U/L; normal range 50.0–310.0)	92 (63–116.5)	72 (47.5–116)	–0.941	.347
Increased	1 (3.2)	2 (6.5)		
Decreased	3 (9.7)	9 (29)		
Lactate dehydrogenase (U/L; normal range 120.0–250.0)	193 (165.5–221)	274 (245.5–297)	–4.057	<.001**
Increased	2 (6.5)	21 (67.7)		
Glucose (mmol/L; normal range 3.9–6.1)	7.79 (6–12.2)	5.2 (4.7–6)	–2.91	.004**
Increased	22 (71)	8 (25.8)		
Decreased	1 (3.2)	1 (3.2)		
Infection-related biomarkers				
C-reactive protein (mg/L; normal range 0.0–5.0)	2.5 (1.3–4.6)	35 (19.5–59.6)	–4.494	<.001**
Increased	7 (22.6)	28 (90.3)		

* *p* < .05.

** *p* < .01.

**Figure 1.**
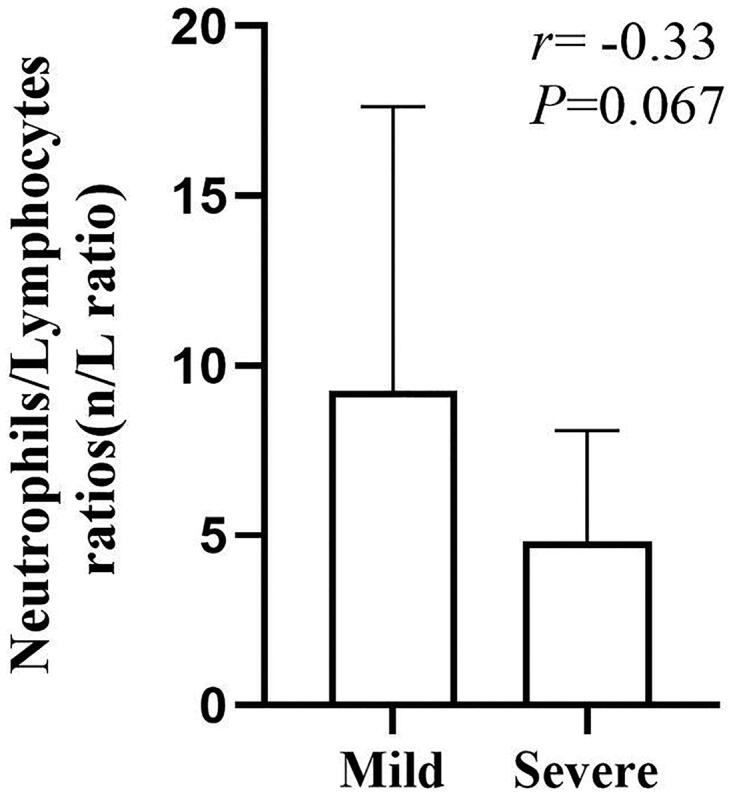
The correlation between the neutrophils/lymphocytes ratio and the severity of the disease.

**Figure 2.**
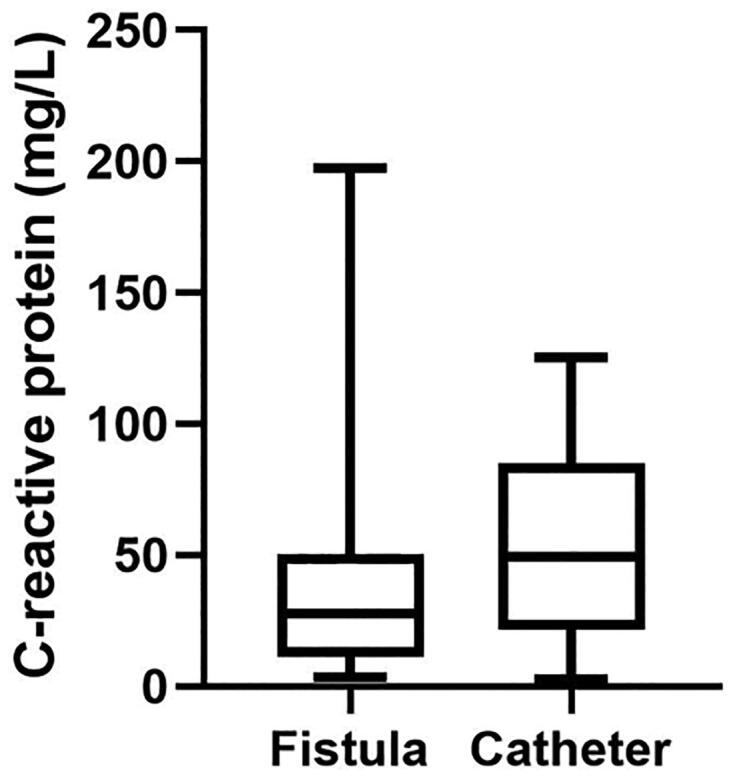
The correlation between the vascular pathway and severity of inflammation.

Compared with mild to severe patients, there was no significant correlation between the neutrophils/lymphocytes ratio and the severity of the disease (Figure 1). There was no significant difference on severity of inflammation between Fistula and Catheter groups (*p*> .05) (Figure 2).

### The imaging, treatment, and outcome of HD patients with COVID-19

**Figure 3.**
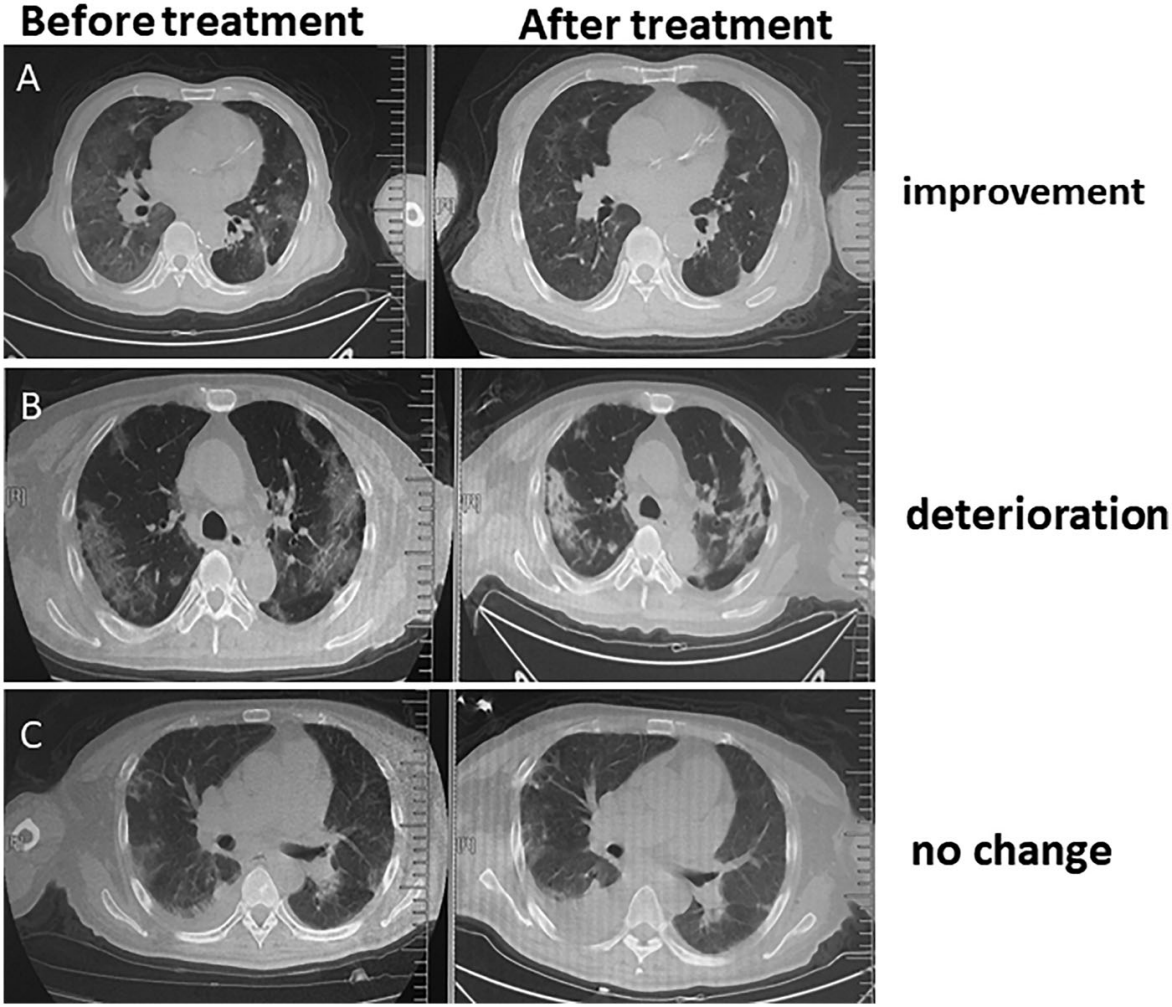
Typical CT scanning imaging features for HD patients infected with COVID-19 before and after treatment. (A) Improvement; (B) deterioration; (C) no change.

All patients underwent chest CT examination. These results displayed that all patients had imaging features of typical COVID-19 infection including multiple mottling and ground glass opacity in single or both lungs at admission (Figure 3).

During hospitalization, all patients were treated with oxygen inhalation by noninvasive mask, 9.7% of them were treated with high flow oxygen while the rest were treated with medium flow oxygen. 90.3% received antihypertensive treatment and the depressor included ACEI, CCB, ARB, and β-blocker. 83.9% of patients received antibiotic treatment which included moxifloxacin, cefoperazone sodium and sulbactam sodium, ceftriazole, cefoxitin, rocephin, ceftazidime, and levofloxacin. These antibiotics can control some atypical pathogens and most common pathogens. Only 12.9% of patients were treated with combination therapy, and the rest were given signal therapy with duration of antibiotic therapy for 3–17 days. 54.8% were given antiviral therapy and the antiviral drugs including abider, oseltamivir, ribavirin, lopinavir, and ritonavir combination. The antiviral drugs were given by single (19.4%), double (77.4%), and triple use (3.2%) with course of treatment for 2–17 days. In addition, the HD patients also received other treatment including glucocorticoid (6.5%), albumin (9.7%), and globulin (9.7%) (Table 4).

**Table 4.** The treatment and outcome of HD patients infected with COVID-19 on admission to hospital.

Treatment	
Mechanical ventilation	31 (100)
Noninvasive (high flow)	3 (9.7)
Noninvasive (face mask)	28 (90.3)
Glucocorticoids	2 (6.5)
Intravenous immunoglobulin	3 (9.7)
Intravenous albumin	3 (9.7)
Antibiotic treatment	26 (83.9)
Levofloxacin injection	5 (16.1)
Levofloxacin injection + moxifloxacin	3 (9.7)
Levofloxacin + ceftezole	1 (3.2)
Moxifloxacin	9 (29)
Rocephin	2 (6.5)
Cefoperazone sodium and sulbactam sodium	4 (12.9)
Ceftazidime	1 (3.2)
Cefoxitin	1 (3.2)
Antiviral treatment	17 (54.8)
Abider + oseltamivir + lopinavir and ritonavir combination	1 (3.2)
Abider + oseltamivir	3 (9.7)
Abider + lopinavir and ritonavir combination	2 (6.5)
Abider + ribavirin	1 (3.2)
Oseltamivir + ribavirin	1 (3.2)
Oseltamivir	3 (9.7)
Lopinavir and ritonavir combination	4 (12.9)
Abider	2 (6.5)
Antibiotic treatment + antiviral treatment	15 (48.4)
Antihypertensive therapy	28 (90.3)
	Patients (*n* = 31)
Comorbid conditions	
Any	18 (58.1)
ARDS	8 (25.8)
Acute heart failure	7 (22.6)
Shock	5 (16.1)
Gastrointestinal hemorrhage	5 (16.1)
Septicemia	1 (3.2)
Cardiac arrest	1 (3.2)
CT finding	
Oblivious improvement	2 (6.5)
Slightly improvement	13 (41.9)
No difference	3 (9.7)
Deterioration	4 (12.9)
No review	9 (29)
Time of admission remission	
No remission	11 (35.5)
Remission (days)	20 (64.5)
1–4	4 (12.9)
5–9	10 (32.3)
10–14	5 (16.1)
15	1 (3.2)
Clinical outcome	
Remained in hospital	27 (87.1)
Discharged	2 (6.5)
Died	2 (6.5)

As of 16 March 2020, 71% of patients had reexamining chest CT scans. Compared with the lung lesions from CT on admission, the outcome of patients included improvement (54%), deterioration (16%), and no change (30%) (Figure 2, Table 4). 58.1% of patients had organ function injury including ARDS (25.8%), acute heart failure (22.6%), and septic shock (16.1%), etc. 64.5% of the patients had remission, and 35.5% of them had no remission with 6.7% deaths. 87.1% of patients are still in the hospital and 6.7% of them have been discharged to date (Table 4).

## Discussion

This is a retrospective study about the epidemiology and clinical features of 31 HD patients with COVID-19, who are transferred from different dialysis centers in Wuhan. Therefore, the collecting data of the above patients represent typical clinical characteristics of HD patients with COVID-19 in China.

In the study, the vascular access of HD patients with COVID-19 was mainly for internal fistula which is in line with common HD patients whereas the ratio of semi-permanent dialysis tube for vascular access is higher than other HD patients probably due to old age, worse vascular condition, more underlying diseases and more critical conditions. As we know, the frequency and duration of dialysis in ordinary HD patients are three times a week and 4 h each time, respectively [5,6]. Therefore, the duration of each dialysis and frequency of dialysis in HD patients with COVID-19 are shorter in outpatient, but there is no difference on the dialysis vintage and dialysis frequency in the hospital. The reason of which is to reduce contact period and avoid cross infection between patients and staff members after the outbreak of COVID-19.

In this study, we observed that there are far more men than women with COVID-19 in HD patients who are in line with the previous study on general population. This is due to the fact that the number of women in HD is lower than the men (13:18), and the women have sex hormones and X chromosome, which can regulate adaptive and innate immunity [7]. In addition, most HD patients infected with COVID-19 were mainly the elderly and those with other chronic diseases besides renal failure which is in line with the previous study, but the average age and odds of chronic disease were higher [7]. This may be because the HD patients had older average age, more complications, and weaker immunity than the general population [6]. Moreover, the epidemiology, symptoms, sign, and oxygen saturation of HD patients were similar to reported common patient infected with COVID-19 which further demonstrated COVID-19 as a human infectious disease and the main clinical features of COVID-19 infection were fever, cough, shortness of breath, and hypoxia. It is noteworthy that HD patients had lower rates of fever and cough than common people that may attribute to HD patients of older age, more sluggish on thermoregulatory center and cough response. The results displayed that the HD patients had lower CPIS than the general population which prompted critical cases with respiratory failure be arranged to another designated hospital to be treated before admission. In addition, the study displayed the low positive rate of nucleic acid test for COVID-19 for the reason that early in the outbreak of COVID-19, the collected samples are unqualified, single site, and limited tests.

In our study, some HD patients have a slight increase in the number of leukocytes and neutrophils with no oblivious difference on platelets which is in line with the previous study. The increase of leukocytes and neutrophils in some people may be induced by bacterial infection and stress. In addition, HD patients have more serious anemia and coagulation disorders than other people. The reasons of which include insufficient erythropoietin secretion, inadequate iron intake, hemorrhage, chronic inflammation, vascular endothelial damage, and hemodynamic disorder induced by renal failure, dialysis, and infection [6]. Especially noteworthy, the result displayed COVID-19 infection and long-term dialysis can reduce lymphocyte count [8]. This implied that the immune function of dialysis patients is impaired and 2019-nCoV may deplete considerable immune cells and suppress cellular immune function of the body. Therefore, destroying lymphocyte is an important factor that causes the deterioration of patients and low lymphocyte count is a useful reference index in the diagnosis of COVID-19 infection. Moreover, there was no significant correlation between the neutrophils/lymphocytes ratio and the severity of the disease. Therefore, neutrophils/lymphocytes ratio does not reflect the severity of the HD patients with COVID-19.

The study displayed that in comparison with other people with COVID-19, the HD patients have lighter hepatic injury which indicates that hemodialysis (HD) is helpful to inhibit inflammatory storm and eliminate harmful substances [1,9]. In addition, the HD patients had more serious myocardial injury as it displayed higher expression of myocardial injury enzyme than other people. The reason of which is that most HD patients have more underlying heart disease, stronger severe infection, heart failure and inflammatory response, and lower immunity than the general population. Furthermore, the HD patients have higher expression of BUN, Cr, BNP, uric acid, and lower expression of ALB than the previous reported common population with COVID-19. This is induced by renal failure that lead to dysfunction of secreting Cr, BUN and urine acid, obstacle of nutrient absorption, and inadequate dialysis [6]. It is worth noting that HD patients have higher susceptibility and more serious hyperkalemia, low calcium and high phosphorus than other population with COVID-19 and common HD patients. Some factors that caused such are long-term inadequate dialysis, hyperlysis caused by infection, input stock blood, and side effects of drugs in HD patients with COVID-19.

PCT is often used as a sensitive marker to evaluate bacterial infection and infection degree in clinic. The result displayed that HD patients have higher bacterial infection rate and more severe infection degree than the general population with COVID-19. This can be due to lower immunity, more underlying disease, and old age of HD patients than other population. Moreover, the HD patients have stronger inflammation as demonstrated with higher expression of CRP and ESR than other population owing to the HD patients that have more serious bacterial infection and are in chronic inflammatory state for a long time.

During hospitalization, the HD patients were treated with standard antihypertensive treatment, strong anti-infection, and active antiviral treatment which is in line with previous study.

In addition, the result displayed that the HD patients had higher mortality rates, more complications, lower discharge, and improvement rates than other infected people that may be because HD patients had less timely treatment, more severe infections and inflammation, less immunity and more strained medical resources. Therefore, it is crucial to conduct early identifying and timely treating critical cases.

There are several limitations of research. First, only 31 HD patients with clinic confirmed COVID-19 were involved; second, the collecting data were acquired from a single center; third, the patients in this hospital are mainly mild and severe, while the patients in critical condition are all admitted to other designated hospitals with ICU. Fourth, more detailed information of HD patients, especially regarding dynamic change of laboratory data and clinical outcomes of HD patients after discharge, was unavailable for analysis; however, the study provided an early evaluation of the clinical characteristics of HD patient with COVID-19.

In conclusion, compared with the general population, the HD patients are susceptible to COVID-19 infection, especially older men and those with other underlying diseases. Likewise, HD patients have more severe infection and inflammation with less symptoms and worse outcome. COVID-19 infection can cause dialysis patients lower immunity, stronger inflammation, malnutrition, and internal environment disorder. Neutrophils/lymphocytes ratio does not reflect the severity of the HD patients with COVID-19.
